# Association between life satisfaction, sleep disturbance and mental health problems among the migrant older adults with children: a conditional process analysis based on per capita bedroom area

**DOI:** 10.1186/s12877-025-05957-y

**Published:** 2025-05-08

**Authors:** Mingli Pang, Jieru Wang, Hexian Li, Guangwen Liu, Xiaoxu Jiang, Jing Xu, Shixue Li, Fanlei Kong

**Affiliations:** 1 Department of Social Medicine and Health Management, School of Public Health, Cheeloo College of Medicine, Shandong University, Jinan, 250012 China; 2 NHC Key Lab of Health Economics and Policy Research (Shandong University), Jinan, 250012 China; 3 Center for Health Management and Policy Research, Shandong University (Shandong Provincial Key New Think Tank), Jinan, 250012 China; 4 National Institute for Health Innovation, School of Population Health, The University of Auckland, Auckland, 1023 New Zealand

**Keywords:** Mental health problems, Life satisfaction, Sleep disturbance, Per capita bedroom area, Migrant older adults following children, Conditional process analysis

## Abstract

**Background:**

The relationship between life satisfaction and mental health problems had been confirmed in many existed studies, but the underlying mediating and moderating effect of sleep disturbance and per capita bedroom area (PCBA) behind this association had never been identified. This study aimed to explore the mediating role of sleep disturbance and the moderating effect of PCBA on the association between life satisfaction and mental health problems.

**Methods:**

Multistage cluster random sampling method was used to select the participants and finally 613 migrant older adults with children (MOAC) were included in the survey. A conditional process model was performed to examine the relationship between life satisfaction and mental health problems, as well as the mediating effect of sleep disturbance and the moderating effect of PCBA.

**Results:**

Life satisfaction was negatively associated with mental health problems, and sleep disturbance could mediate their association. Furthermore, the direct effect of life satisfaction on mental health problems and the indirect effect of sleep disturbance in the relationship were moderated by PCBA, but it only moderated the direct effect and the second indirect link (sleep disturbance-mental health problems) of the mediating effect. Both these two effects were stronger for MOAC with a low level of PCBA.

**Conclusions:**

Life satisfaction had negative effect on mental health problems. PCBA moderated the direct effect of life satisfaction on mental health problems and the mediating effect of sleep disturbance on mental health problems. For MOAC with a low level of life satisfaction and high sleep disturbance, particularly those with a low level of PCBA, targeted implication for the community, family members and MOAC were proposed to improve the mental health of MOAC.

## Background

With the fast development of aging and urbanization over the past decades, the migrant population of China reached 376 million in 2020 [1]. Meanwhile, the number of migrant older adults in China also increased rapidly since 2000 and reached 13.4 million in 2015, with the annual average growth rate was 6.6% [2]. Among these migrant older adults, some of them migrated following their children to big cities to take care their grandchildren or reunite with their family, they were defined as migrant older adults with children (MOAC) [3]. MOAC could participate in caring grandchildren, food cooking and household chores to reduce the burden of their children, thus playing an important role in the maintenance of family function and harmonious society. However, the migrant older adults were also the vulnerable populations, since they were suffered from lower healthcare utilization [4, 5], social integration problems [6] and mental health problems [7, 8]. Therefore, to carry out the research on the mental health problems of the MOAC could help identify modifiable risk factors that affected mental health and promoted the improvement in mental health status of MOAC; it was conducive to promoting MOAC’s sense of self-worth and better integration into family and society.

Life satisfaction referred to a subjective assessment of the quality of one’s life [9]. which had been confirmed to be one important determinants of mental health problems [10]. A study among the young adults showed that low life satisfaction was associated with psychological problems [11]. Meanwhile, a study among older adults in US found that most domains of life satisfaction were associated with psychological outcomes [12] and a study among university students in Korea illustrated that better life satisfaction was associated with a lower risk of depression [13]. Thus, the association between life satisfaction and mental health problems did exist, yet the underlying mediating and moderating mechanisms behind this association had not been identified, not mentioned in the MOAC group.

Sleep disturbance was common in older adults [14], which had been confirmed as a risk factor for multiple mental health problems [15–18], and was usually used as an umbrella term to refer to a variety of sleep problems including sleep disorders, insomnia, hypersomnia, poor sleep quality, and inadequate sleep duration [19]. Furthermore, many studies had clarified the effect of life satisfaction on sleep disturbance [20–23], a study among the middle school students in China showed that life satisfaction was negatively correlated with sleep quality [20]. Accordingly, we conjectured that sleep disturbance may be an intermediate process from life satisfaction to mental health problems. To date, the association between life satisfaction and mental health problems via sleep disturbance remained unexplored, let alone such association among the MOAC.

Per capita bedroom area (PCBA) represented the size of the bedroom of MOAC in this study. Previous study showed that housing situation was associated with people’s psychological health [24] and the household crowding was found to be a major risk factor for epidemic disease [25]. Moreover, a study in New Zealand showed the significant association between the housing circumstances and psychological distress [26]. However, no study had clarified the association between PCBA and mental health problems, not mentioned the moderated effect of PCBA between life satisfaction and mental health problems, as well as sleep disturbance and mental health problems in a moderated mediation model.

**Fig. 1:**
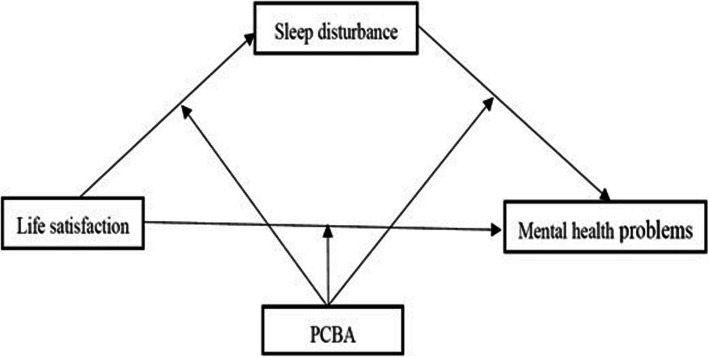
The proposed theoretical moderated-mediation model. Note: the first half indirect effect referred to the line from life satisfaction to sleep disturbance; the second half indirect effect referred to the line from sleep disturbance to mental health problems

In summary, the first aim of this study was to test whether sleep disturbance mediated the relationship between life satisfaction and mental health problems, the second aim was to investigate the moderated role of PCBA (including the moderated effect on the direct effect in the relationship between life satisfaction and mental health problems, as well as the indirect effect between life satisfaction and mental health problems through sleep disturbance), the third aim was to recommend implications to reduce mental health problems among MOAC and to promote a better life for MOAC in the migrated city. Based on the social ecological model of sleep and social ecological model of sleep and health [27, 28], the conceptual framework of the study was shown in Fig. 1, and three hypotheses were proposed as follow:

- Hypothesis 1. Life satisfaction would be negatively and directly associated with mental health problems.
- Hypothesis 2. Sleep disturbance would mediate the relation between life satisfaction and mental health problems.
  Hypothesis 3. PCBA would moderate the effects of life satisfaction and sleep disturbance on mental health problems.

## Methods

### Participants

A total of 613 MOAC was selected in Weifang City, China, in August 2021. According to the 7th National census of Weifang City, the total household population of the city was 9.39 million and the population aged 60 and above was 2.04 million, account for 21.7% of the total population, the migrant population in the city was 2.38 million [29].

Multistage cluster random sampling was employed to select the participants. Firstly, four districts were selected as the primary sampling units (PSUs) based on the economic development and geographical location of Weifang city. Secondly, one sub-district from each PSUs were randomly selected as secondary sampling units (SSUs). Thirdly, one community were randomly selected from each of the SSUs. The migrant population aged 60 years or above in the selected communities who came to live in Weifang with their children more than 3 months constituted the whole sample.

The trained investigators conducted face-to-face interviews with each participant for approximately 20 minutes. Initially, 616 MOAC were selected and interviewed. However, three participants were excluded as they answered their questionnaires without logical or incompletely. Ultimately, a total of 613 participants were included in the database.

### Assessment and measurements

#### Mental health problems

Mental health problems was assessed by the Depression Anxiety Stress Scale (DASS- 21) [30, 31], it was proposed by P. F. Lovibond and S. H. Lovibond [32]. It included three subscales-depression, anxiety and stress, totally 21 items, this scale was rated by a 4-point scale (0–3) and the scores of the three subscales and total scale were calculated, with higher scores indicating higher level of negative mental status [33]. The Cronbach's alpha coefficient was 0.865 for DASS- 21 in this study.

#### Life satisfaction

The Satisfaction with Life Scale (SWLS) was employed to measure life satisfaction, this scale was developed by Diener [34]. It comprised of 5 items and the options for each item ranged from 1 (“strongly disagree”) to 7 (“strongly agree”) and the total score of the scale ranged from 5 to 35 [35]. The Cronbach's alpha coefficient was 0.942 for SWLS in this study.

#### Sleep disturbance

The Pittsburgh Sleep Quality Index (PSQI) [36] was used to measure the sleep disturbance [37] of the participants in the past month before the survey. It consisted of 19 self-reported items and these items could be divided into seven components. Each component was weighted equally on a scale of 0–3. The PSQI total score ranged from 0 to 21. Individuals with higher scores indicate poorer overall sleep quality [36], meant high sleep disturbance, it was appropriate for use among older adults [38, 39]. The Cronbach's alpha coefficient was 0.773 for PSQI in this study.

#### PCBA

PCBA was measured by the question: “Your per capita bedroom area?”, the answer was numerical. The higher numeric, indicated the larger PCBA.

#### Covariates

Covariates included the following parts: gender (male, female) [40], age (60 ~ 65, 66 ~ 70, 71 ~ 80, > 80) [41], marital status (married, single/divorced/separated/widowed) [42], Hukou (rural, urban), education level (not educated, primary school, junior high school, high school/technical secondary school, college degree and bachelor degree or above) [43], employment status (currently employed, retired, unemployed) [44], monthly household income (the first quartile (Q1), the second quartile (Q2), the third quartile (Q3), and the fourth quartile (Q4)) [43]. Q1 was the poorest and Q4 was the richest, time since migration (under five years, five years or above) [45] and chronic disease (without chronic disease, a chronic disease, two chronic diseases, three chronic diseases or above) [43], and the above factors had been confirmed associated with mental health problems.

### Statistical analyses

All statistical analyses were performed by using SPSS version 24.0 (SPSS Inc., Chicago, IL, USA). Firstly, descriptive analysis, frequencies and percentages were employed to illustrate the basic characteristics of MOAC. Secondly, Pearson correlation analysis was used to test the correlations of life satisfaction, sleep disturbance, PCBA and mental health problems. Thirdly, PROCESS v3.5 for SPSS developed by Hayes [46] was employed to verify mediation and the moderated mediation hypotheses. The mediating role of sleep disturbance was tested by Model 4 in PROCESS. The mediating effect was deemed statistically significant if the 95% confidence intervals (CI) of the indirect effect did not contain 0. Fourthly, we introduced PCBA as the moderator variable into the model and tested the moderating role of PCBA in possible direct and indirect effects of life satisfaction on mental health problems by using Model 59. Similarly, the moderated mediation effect was approved statistically significant if the 95% CI of the interaction did not contain 0. Both models had the same independent, dependent, and mediating variables, life satisfaction, mental health problems, sleep disturbance, respectively, except for PCBA, which was included as a moderating variable in the moderated mediation model. All the regression coefficients calculated by the Model 4 and Model 59 were both controlled for gender, age, marital status, education level, job before retire, monthly household income, time since migration and chronic disease. The bootstrap sample size was set at 5000, the CI was 95%, and all continuous variables were meant centering. Statistical significance was defined as *p*-value < 0.05.

## Results

### Characteristics of the participants

The basic information of MOAC were shown in Table 1. A total of 613 MOAC participated and filled in the questionnaire effectively, among them, 73.08% were female, 55.79% were aged 60 ~ 65, 87.93% were married, 85.64% were from rural areas, 30.18% were educated in primary school, 70.80% were unemployed, 53.83% reported under five years since migration and 57.26% without chronic disease. All above variables were controlled as covariates in the moderated mediation model.

**Table 1:** Characteristics of the sample (*N* = 613)

Variable	*n*	%
**Total**	613	
**Sex**		
Male	165	26.92
Female	448	73.08
**Age**		
60 ~ 65	342	55.79
66 ~ 70	171	27.90
71 ~ 80	80	13.05
> 80	20	3.26
**Marital status**		
Married	539	87.93
Single/divorced/separated/widowed	74	12.07
**Hukou**		
Rural	525	85.64
Urban	88	14.36
**Educational level**		
Not educated	161	26.26
Primary school	185	30.18
Junior high school	158	25.77
High school/technical secondary school	91	14.85
College degree, bachelor degree or above	18	2.94
**Employment status**		
Currently employed	53	8.65
Retired	126	20.55
Unemployed	434	70.80
**Monthly household income**		
Q1	154	25.12
Q2	152	24.80
Q3	153	24.96
Q4	154	25.12
**Time since migration**		
Under five years	330	53.83
Five years or above	283	46.17
**Chronic disease**		
Without chronic disease	351	57.26
A chronic disease	170	27.73
Two chronic diseases	70	11.42
Three chronic diseases or above	22	3.59

Note: Q1: the first quartile, Q2: the second quartile, Q3: the third quartile, Q4: the fourth quartile

Q1 was the poorest and Q4 was the richest

### Descriptive analysis and correlation test of main variables

Table 2 showed the mean, standard deviations, and Pearson correlations of the main variables. Life satisfaction was negatively associated with mental health problems (*r* = − 0.39, *P* < 0.01) and sleep disturbance (*r* = − 0.27, *P* < 0.01), sleep disturbance was positively associated with mental health problems (*r* = 0.39, *P* < 0.01). Meanwhile, PCBA was negatively linked with mental health problems (*r* = − 0.11, *P* < 0.01) and positively linked with life satisfaction (*r* = 0.08, *P* < 0.05), while the correlation between sleep disturbance and PCBA was not statistically significant.

**Table 2:** Correlations for the main variables

Variable	Mean (SD)	1	2	3	4
1. Mental health problems	7.77 (10.64)	1			
2. Life satisfaction	27.87 (5.58)	− 0.39**	1		
3. Sleep disturbance	4.29 (3.60)	0.39**	− 0.27**	1	
4. Per capita bedroom area	10.76 (5.25)	− 0.11**	0.08*	− 0.02	1

Note: *SD* Standard deviation

^**^P < 0.01

^*^P < 0.05

### Mediating effect analysis

Table 3 showed the result of mediating effect analysis. After controlling the covariates, the total effects model showed that life satisfaction was negatively associated with mental health problems (Coefficient = − 0.73, t = − 10.14, *P* < 0.001). As for mediation test, life satisfaction was negatively related to sleep disturbance (Coefficient = − 0.15, t = − 6.20, *P* < 0.001), and sleep disturbance was positively associated with mental health problems (Coefficient = 0.93, t = 8.25, *P* < 0.001), life satisfaction was negatively associated with mental health problems (Coefficient = − 0.59, t = − 8.34, *P* < 0.001). The bias-corrected percentile bootstrap results with 10,000 re-samples showed the 95% CI around direct effect (Effect = − 0. 59, 95%CI, − 0.73, − 0.45) and indirect effect (Effect = − 0.14, 95%CI, − 0.23, − 0.08) did not contain zero (Table 4), implied the association between life satisfaction and mental health problems was partially mediated by the sleep disturbance.

**Table 3:** Mediation analysis of sleep disturbance

Variable	Model 1 (Mental health problems)		Model 2 (Sleep disturbance)		Model 3 (Mental health problems)	
	**Coefficient t**		**Coefficient t**		**Coefficient t**	
Life satisfaction	− 0.73	− 10.14***	− 0.15	− 6.20***	− 0.59	− 8.34***
Sleep disturbance					0.93	8.25***
R^2^	0.17		0.13		0.25	
F	12.37		9.00		18.69	

Note: adjusted for gender, age, marital status, hukou, education level, employment status, monthly household income time since migration and chronic disease

^***^P < 0.001

**Table 4:** Bootstrap results for effect of life satisfaction on mental health problems

Item	Effect	SE	LLCI	ULCI
Direct effect	− 0.59	0.07	− 0.73	− 0.45
Indirect effect	− 0.14	0.04	− 0.23	− 0.08

Note: adjusted for gender, age, marital status, hukou, education level, employment status, monthly household income time since migration and chronic disease

*LLCI: *Lower limit confidence interval 95%, *ULCI:* Upper limit confidence interval 95% (bias-corrected bootstrap confidence intervals), *SE* Standard error.

### Moderated mediation analyses

The moderated mediation analyses were showed in Table 5. Controlled the covariates, the model with sleep disturbance as outcome (F = 7.567, *P* < 0.05) and dependent variable model (F = 16.578, *P* < 0.05) were both statistically significant. Two significant interactions were analyzed to test the moderating role of PCBA. In the sleep disturbance as outcome model, life satisfaction was negatively associated with sleep disturbance (Coefficient = − 0.152, t = − 6.073, *P* < 0.001), however, the effect of PBCA and the interaction (life satisfaction * PCBA) were not statistically significant (both *P* > 0.05), implied that PCBA didn’t moderate the first indirect link (life satisfaction -sleep disturbance) of the mediating effect. In the dependent variable model, the interaction effect of life satisfaction and PCBA on mental health problems were significantly (Coefficient = 0.028, t = 2.172, *P* = 0.030), meanwhile, the interaction effect of sleep disturbance and PCBA on mental health problems were also significantly (Coefficient = − 0.052, t = − 2.583, *P* = 0.010). These findings illustrated that PCBA was as a moderator in the association between life satisfaction and mental health problems, sleep disturbance and mental health problems.

**Table 5:** Regression results of the moderated mediation model (model 59)

Outcome Variable				
**Outcome variable: sleep disturbance**	**Coefficient**	**SE**	**t**	***P***
Constant	− 4.568	1.284	− 3.556	< 0.001
Life satisfaction	− 0.152	0.025	− 6.073	< 0.001
PCBA	− 0.025	0.027	− 0.931	0.352
Life satisfaction * PCBA	0.002	0.005	0.361	0.718
R^2^	0.131			
F	7.567			
**Outcome variable: Mental health problems**	**Coefficient**	**SE**	**t**	***P***
Constant	7.292	3.532	2.065	0.039
Life satisfaction	− 0.575	0.070	− 8.208	< 0.001
sleep disturbance	0.895	0.111	8.043	< 0.001
PCBA	− 0.185	0.073	− 2.517	0.012
Life satisfaction * PCBA	0.028	0.013	2.172	0.030
Sleep disturbance * PCBA	− 0.052	0.020	− 2.583	0.010
R^2^	0.280			
F	16.578			
**Conditional direct effect analysis at different values of PCBA (M ± SD)**	**Effect**	**SE**	**LLCI**	**ULCI**
M- 1SD (5.506)	− 0.722	0.096	− 0.911	− 0.533
M (10.760)	− 0.575	0.070	− 0.713	− 0.438
M + 1SD (16.014)	− 0.428	0.098	− 0.621	− 0.235
**Conditional indirect effect analysis at different values of PCBA (M ± SD)**	**Effect**	**Boot SE**	**Boot LLCI**	**Boot ULCI**
M- 1SD (5.506)	− 0.187	0.067	− 0.331	− 0.072
M (10.760)	− 0.136	0.036	− 0.215	− 0.072
M + 1SD (16.014)	− 0.090	0.037	− 0.173	− 0.029

Note: Bootstrap sample size = 5000.  *: denoted the product of two variables, representing the interaction term.

*SE:* Standard error, *LLCI:* Lower limit confidence interval 95%, *ULCI:* Upper limit confidence interval 95%, *SD:* Standard deviation.

If the number of decimal places is the same as in the previous table, some data cannot be fully displayed, so in this table, the number of decimal places is kept to three decimal places

Furthermore, the significant moderated mediation models were tested by analyzing the direct and indirect effects of life satisfaction on mental health problems at different levels of PCBA. PCBA was divided into the high-level group (M + 1SD), moderate (Mean), and low-level group (M- 1SD). The 95% CI in conditional direct and conditional indirect effects showed that the low-level group and the high-level group both did not include zero. The direct effect of life satisfaction on mental health problems and the effect of sleep disturbance on mental health problems were stronger at the low level of PCBA.

**Fig. 2:**
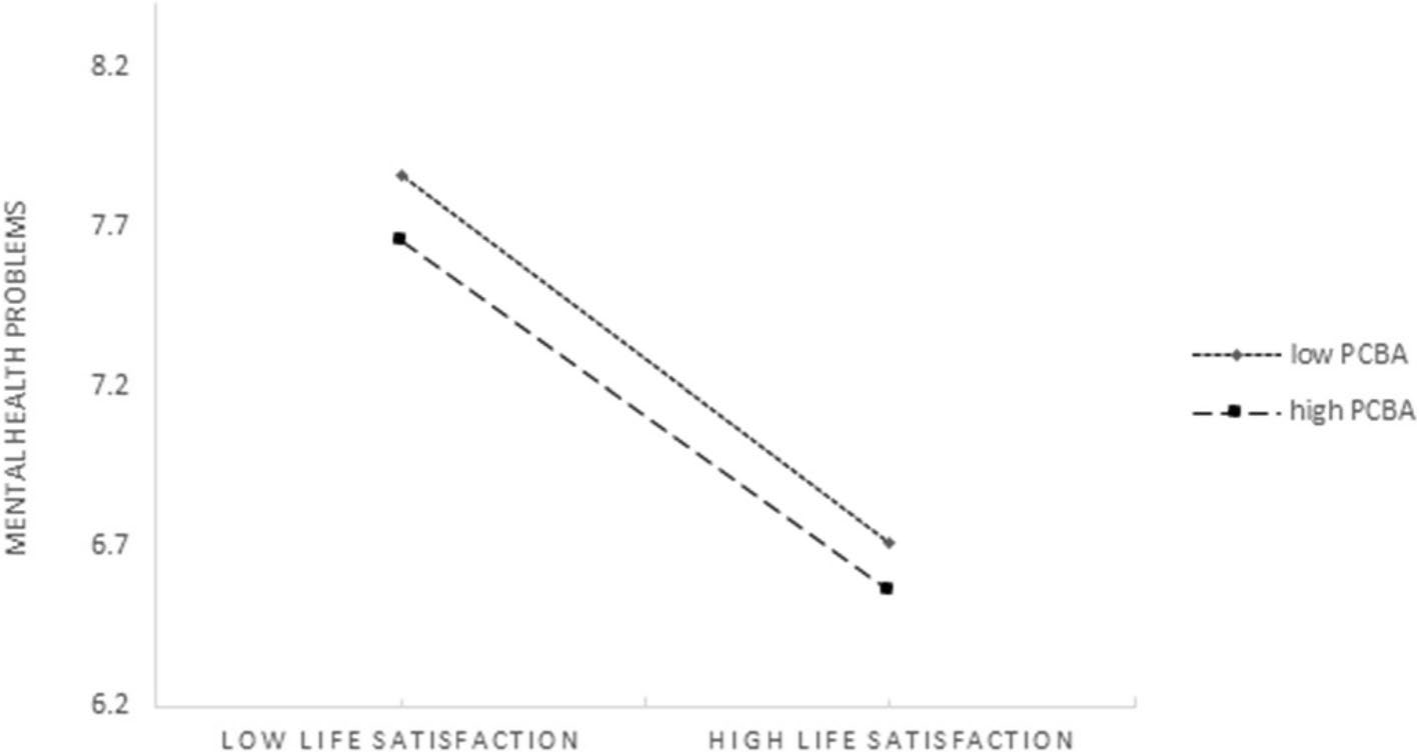
Conditional effects of PCBA on association between life satisfaction and mental health problems

**Fig. 3:**
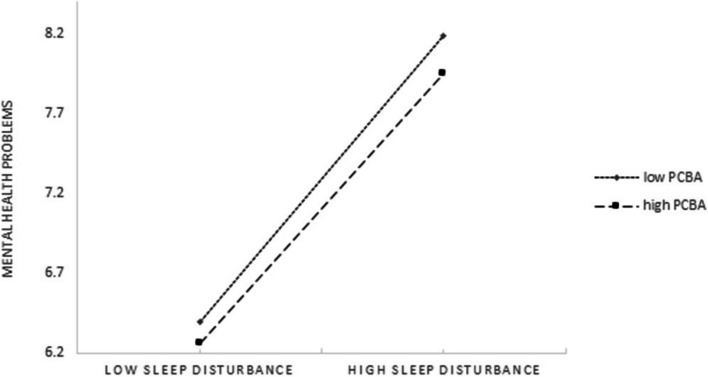
Conditional effects of PCBA on association between sleep disturbance and mental health problems

The moderating effect could be visualized in Figs. 2 and 3. As shown in Fig. 2, whether high level or low level of PBCA among the MOAC, as the life satisfaction increased, mental health problems decreased significantly. The lower level of PBCA, the more obvious the trend (larger the slope). As shown in Fig. 3, at low levels of sleep disturbance, mental health problems remained low regardless of PBCA. However, when sleep disturbance was high, a low level of PCBA leaded to the higher increase in mental health problems. Furthermore, the direct effect of life satisfaction on mental health problems and the second indirect link (life satisfaction -sleep disturbance) of the mediating effect were more significant for participants with low level of PCBA.

## Discussion

This study examined the mediating and moderating mechanisms of the sleep disturbance and PCBA underlying the relationship between life satisfaction and mental health problems via a moderated mediation model among the MOAC in Weifang city, China. In this study, the negative association between life satisfaction and mental health problems was found, a partial mediation role of sleep disturbance was also observed in the above association. Furthermore, PCBA moderated the direct effect of life satisfaction on mental health problems, as well as the second half of the indirect effect from sleep disturbance to mental health problems. To be specific, when life satisfaction was low, a low level of PCBA would lead to the direct increase of mental health problems; when life satisfaction was low, a low level of PCBA would result in the indirect increase of mental health problems due to sleep disturbance.

This study found the life satisfaction was negatively associated with the mental health problems, which confirmed the Hypothesis 1 and was consistent with the previous studies [47–49]. In detailed, the higher the life satisfaction, the lower the mental health problems. It was widely acknowledged that the life satisfaction was an individual's comprehensive judgment of their own life, which could affect the individual's emotional experience [50]. The high level of life satisfaction might decrease the negative effects of stress exposure on the individual, due to it could help the individual to recover from negative life events [51] and result in a better mental health.

This study illustrated that the association between life satisfaction and mental health problems was partially mediated by sleep disturbance, which confirmed the Hypothesis 2 and also explained how life satisfaction could indirectly affect mental health problems. None study had illustrated the mediating effect of sleep disturbance on the relationship between life satisfaction and mental health problems, yet Parkerson et. al’s study showed that the high life satisfaction could lead to more sleep among first-year medical students [49], while Eleftheriou et. al’s study found that the more sleep disturbance, the more mental health problems [52].

Based on the moderated mediation analyses, PCBA was found could moderate the direct effect of life satisfaction on mental health problems and the second half of the indirect effect from sleep disturbance to mental health problems, thus partly supported the Hypothesis 3. Existed studies had illustrated that household overcrowding was harmful to individuals'mental health problems [53, 54], yet no study had focused on the association between PCBA and mental health problems, let alone clarifying the moderating effects of PCBA on the relationship between life satisfaction/sleep disturbance and mental health problems. The findings could be explained as follows. Firstly, PCBA could moderate the direct effect of life satisfaction on mental health problems. The negative direct correlation between life satisfaction and mental health problems was higher among those with low PCBA than the high PCBA (as shown in Fig. 2). Previous research illustrated the house mainly represented a safe place for individuals [55], low level of PCBA could not offer an adequate private space for the MOAC to relax and adjust their mood, which furtherly increase low life satisfaction’s negative effect on mental health problems. Secondly, PCBA moderated the indirect link (sleep disturbance-mental health problems) of life satisfaction on mental health problems mediated by sleep disturbance. That is, for the MOAC with a low level of PCBA, the negative effect of life satisfaction on mental health problems was higher when the sleep disturbance increased. In detailed, as shown in Fig. 3, higher indirect link (sleep disturbance-mental health problems) was found among MOAC with a low level of PCBA than those with a high level of PCBA. The explanation might be as follow: the MOAC with low life satisfaction had higher levels of negative emotions than those with high life satisfaction, which furtherly lead to the increase of the sleep disturbance symptoms; to make thing worse, the low level of PCBA would not provide an ideal place for sleep, and finally aggravated the negative effect of lower life satisfaction on mental health problems. This result was in line with the previous studies which also found the bedroom condition was associated with the sleep disturbance and mental health problems [56, 57]. Thirdly, the pandemic of the infectious disease made the duration of the people’s staying at home longer, the smaller private PCBA, together with the social distancing measures would lead to more mental health problems.

Based on the results above in this study, targeted implications on the improvement of life satisfaction, sleep quality, PCBA and mental health among the MOAC were given as follow. Firstly, the children of MOAC should pay more attention on the MOAC’s life satisfaction, sleep quality, and mental health problems, communicated more with MOAC and reduced the burden of MOAC in daily life to reduce physical and mental stress. Secondly, the children of MOAC should try their best to provide a larger private bedroom for MOAC. Thirdly, the community should promote the construction of a communication platform for MOAC, build a platform for them to communicate with their peers, reconstruct the social support and social network, improve life satisfaction and mental health of MOAC. Fourthly, MOAC should keep a positive attitude about life, open heart to children and other family members and keep relax to improve life satisfaction, sleep quality and mental health.

For future studies, follow-up study should be conducted to deeply reveal the correlation and influencing mechanism among life satisfaction, sleep disturbances and mental health problems, meanwhile, to further study the dynamic change of mental health problems among MOAC. Secondly, the regional scope of the study could be expanded, thus to study the differences in mental health problems among MOAC in different cities, furthermore, employing multi-group analysis, explored the influence mechanism of life satisfaction, sleep disturbances, PCBA and mental health problems among MOAC.

There were several limitations in this study. Firstly, the reliance on community staffs’ help on participant recruitment might affect the diversity of the sample and introduce potential bias in the selection of participants, despite the researchers’ efforts to ensure the representation of MOAC across different age groups and geographical locations within the region. Secondly, PCBA was completed by self-reporting, and some MOAC could only know the general PCBA information rather than exact PCBA data, furthermore, the level of housing prices in different parts of the city or whether it is school district housing was not taken into account. Thirdly, there were other factors such as social disintegration and lower healthcare utilization that could mediate the relationship between life satisfaction and mental health problems, however, this study only studied the mediating of sleep disturbances on the association between life satisfaction and mental health problems and resulted the scope of the study was not comprehensive enough. Fourthly, some covariates were regrouped on the basis of the original data, and it might produce bias. Lastly, this study was a cross-sectional study, which made a causal relationship was difficult to be predicted, there might existed the path that life satisfaction mediated the association between sleep disturbance and mental health problems.

## Conclusions

To summarize, life satisfaction was negatively associated with mental health problems among the MOAC in Weifang, China; sleep disturbance played a mediating role in the relationship between life satisfaction and mental health problems; the direct effect of life satisfaction on mental health problems and the indirect effect between sleep disturbance and mental health problems were moderated by PCBA. Targeted implication for the community, family members on those MOAC with a low level of life satisfaction, high sleep disturbance and a low level of PCBA, were proposed based on the results above.

## Data Availability

Under reasonable requirements, the data and material of this study can be obtained from the corresponding author. The data are not publicly available due to privacy restrictions.

## Abbreviations

PCBA: Per capita bedroom area
MOAC: Migrant older adults with children
PSUs: The primary sampling units
SSUs: Secondary sampling units
DASS- 21: Depression Anxiety Stress Scale
SWLS: The Satisfaction with Life Scale
PSQI: The Pittsburgh Sleep Quality Index
CI: Confidence interval
Q1: The first quartile
Q2: The second quartile
Q3: The third quartile
Q4: The fourth quartile
SD: Standard deviation
LLCI: Lower limit confidence interval 95%
ULCI: Upper limit confidence interval 95%
SE: Standard error
